# Role of Father–Child Relational Quality in Early Maladaptive Schemas

**DOI:** 10.5812/ijhrba.4381

**Published:** 2012-07-25

**Authors:** Nader Monirpoor, Morteza Gholamyzarch, Mohsen Tamaddonfard, Helen Khoosfi, Ali Reza Ganjali

**Affiliations:** 1 Department of Clinical Psychology, Islamic Azad University, Qom Branch, Qom, IR Iran; 2 Young Researches Club, Rudehen Branch, Islamic Azad University, Rudehen, IR Iran; 3 Young Researches Club, Qom Branch, Islamic Azad University, Qom, IR Iran; 4 Department of Clinical Psychology, Counsulting Center of Shahed University, Shahed University, Tehran, IR Iran; 5 Department of Psychiatry and Clinical Psychology, Zahedan University of Medical Sciences, Zahedan, IR Iran

**Keywords:** Paternal Behavior, Father-Child Relation

## Abstract

**Background:**

Primary maladaptive schemas, which are the basis of high-risk behavior and psychological disorders, result from childhood experiences with significant objects, such as fathers, in different developmental phases.

**Objectives:**

This endeavor examined the role of the father in predicting these schemas.

**Patients and Methods:**

A total of 345 Islamic Azad University students (Qom Branch) who were chosen through convenience sampling completed the Young Schema Questionnaire, the Parental Bonding Instrument, and the Parent–Child Relationship Survey.

**Results:**

A multivariate regression analysis indicated that anumber of aspects of the father–child relationship, including care, emotional interaction, positive affection, the effective relationship, and excessive support, predict particular schemas.

**Conclusions:**

Therefore, these findings suggested that psychotherapists examine the different aspects of the father–child relationship when restructuring schemas.

## 1. Background

After deadlocking on some aspects of cognitive therapy in the treatment of psychological disorders, especially depression, new theories have been presented in recent years. Schema therapy (1), which offersthe concept of the Early Maladaptive Schema (EMS), provides hopeful perspectives in the explanation and formulation of psychological disorders (2). This theory is combined with different cognitive and psychodynamic theories. Schema theories emphasis that EMSs constitute the basis of psychological disorders (3). These schemas result from childhood experiences with important objects in different developmental phases. Schemas, which include cognitions, emotions, physical sensations, and coping strategies, perpetuate psychological disorders by imposing schema-driven actions. Young emphasized that conflicting experiences with parents, sisters, brothers, or peers are one of the most important factors in EMS formation during childhood (4). Parents or early attendants have an important role in the constitution of EMSs. Most theorists and researchers have examined the role of parents, especially mothers, in the creation of these schemas and have established their curative interventions on this basis. Researchers’ clinical experiences with different disorders, especially borderline personality disorder, depression, and other disorders, have shown an effective role of the father. However, previous studies in recent years have further focused on mother–child relationships, and fathers have been thought of as a peripheral person who has little direct effect in the child’s development (5). In fact, different studies have neglected the fathers’ role in the formulation of psychological disorders. Therefore, because of researchers’ experiences and because of the remarkable position of the father in Iranian culture, this study was conducted with the aim of assessing fathers’ roles in the formulation of early maladaptive cognitive schemas.

## 2. Objectives

This study carried out with the aim of assessing fathers’ roles in the explaining of early maladaptive cognitive schemas.

## 3. Patients and Methods

### 3.1. Participants and Plan

In this cross-sectional study, 345 (218 females, 127 males) Islamic Azad University students, Qom Branch (in 2010), were chosen through convenience sampling. 

### 3.2. Measures

The measures used for gathering the data included the following: Young Schema Questionnaire-Short Form (YSQ-SF), Parental Bonding Instrument (PBI)-father form (PBI-FF), and the Parent–Child Relationship Survey (PCRS).

#### 3.2.1.Young Schema Questionnaire-Short Form

The YSQ-SF included 75 items and was created by Young and Brown (6) in order to measure EMSs.This questionnaire measures the following 15 primary maladaptive schemas: 1) Emotional Deprivation, 2) Abandonment/Instability, 3) Mistrust/Abuse, 4) Social Isolation/Alienation, 5) Defectiveness/Shame, 6) Failure, 7) Dependence/Incompetence, 8) Vulnerability to Harm or Illness, 9) Enmeshment/Undeveloped Self, 10) Subjugation, 11) Self Sacrifice, 12) Emotional Inhibition, 13) Unrelenting Standards, 14) Entitlement/Grandiosity, and 15) Insufficient Self Control. The YSQ-SF has been shown to have adequate reliability and validity in Iranian samples. For instance, a research (7) assessed the validity of this instrument with a SCL–25 (8).

#### 3.2.2. Parental Bonding Instrument-Father Form

The PBI-FF included 25 Questions and was developed by Parker et al. (9)in order to measure the views of children about their care by their parents and their excessive support. In Iran, the alpha coefficients for maternal care, paternal care, maternal excessive support, and paternal excessive support were reported as 0.90, 0.90, 0.85,and 0.85,respectively (10).

#### 3.2.3. Parent–Child Relationship Survey

The Parent-Child Relationship Survey (11) included 24 questions designed to evaluate youth’s ideas about theirrelationship with their parents. The instrument subscales include positive affect, father involvement, communication and conversation, and lack of angriness. In a study (12) on 151 girls and boys, its alpha coefficient was reported as 0.94.

### 3.3. Procedure

After selecting the research sample, the Questionnaires were given to the participants, and they were asked to complete the research instruments. The data were analyzed by Pearson correlations and multivariate regressions with the forward method.

## 4. Results

A correlation matrix of the variables is reported in *Table 1*. In the multivariate regression, EMS was entered as a predicted variable and father-related variables were entered as predicting variables.The multivariate regression results are reported in *Table 2*. The results reported in *Table 2* indicate the following: father care and father emotional involvement predicted 20.7% of the emotional deprivation schema variance; father excessive support predicted 0.9% of the abandonment schema variance, 2.4% of the enmeshment-undeveloped self schema variance, and 1.5% of the unrelenting standards schema variance; father excessive support and emotional interaction predicted 9.3% of the mistrust-mistreat schema variance, 11.7% of the social isolation schema variance, and 13.4% of the defectiveness-shame schema variance; positive father’s affection predicted 8.5% of the failure to achieve schema variance and 9.4% of the dependence-incompetence schema variance; father excessive support and positive father’s affection predicted 5.9% of the vulnerability to harm and illness schema variance; father care and excessive support predicted 13.6% of the subjugation schema variance; relationship with father predicted 8.7% of the emotional inhibition schema, and 5.5% of the entitlement-self-centeredness schema; and relationship with father and lack of child’s anger toward father predicted 8.9% of the insufficient self-control/self-discipline schema. In addition, the self-sacrifice schema had no meaningful correlation with father-related variables.

**Table 1.** Correlations Between the Parental Bonding Instrument (PBI), the Parent–Child Relationship Survey (PCRS),and the Early Maladaptive Schema (EMS)

	A ^a^	B ^a^	C ^a^	D ^a^	E ^a^	F ^a^	G ^a^	H ^a^	I ^a^	J ^a^	K ^a^	L ^a^	M ^a^	N ^a^	O ^a^	P ^a^	Q ^a^	R ^a^	S ^a^	T ^a^	U ^a^
A ^a^	-																				
B ^a^	-0.429 ^b^	-																			
C ^a^	0.685 ^b^	-0.359 ^c^	-																		
D ^a^	0.764 ^c^	-0.377 ^c^	0.774 ^c^	-																	
E ^a^	0.711 ^c^	-0.410^c^	0.692 ^c^	0.803 ^c^	-																
F ^a^	0.505 ^c^	-0.358^c^	0.602 ^c^	0.533 ^c^	0.518 ^c^	-															
G ^a^	-0.428 ^c^	0.272 ^c^	-0.3388^a^	-0.435 ^c^	-0.387 ^c^	-0.244 ^c^	-														
H ^a^	-0.106 ^b^	0.108 ^b^	-0.045	-0.099	-0.082	-0.093	0.238 ^c^	-													
I ^a^	-0.226	0.275 ^c^	-0.230 ^c^	-0.243 ^c^	-0.203 ^c^	-0.150 ^c^	0.373 ^c^	0.380 c	-												
J ^a^	-0.203 ^c^	0.258 ^c^	-0.284 ^c^	-0.316 ^c^	-0.231 ^c^	-0.175 ^c^	0.478 ^c^	0.279 ^c^	0.524 ^c^	-											
K ^a^	-0.306 ^c^	0.274 ^c^	-0.319 ^c^	-0.338 ^c^	-0.272 ^c^	-0.239 ^c^	0.569^c^	0.388 ^c^	0.445 ^c^	0.604^c^	-										
L ^a^	-0.219 ^c^	0.162 ^c^	-0.295 ^c^	-0.273 ^c^	-0.259 ^c^	-0.177 ^c^	0.398 ^c^	0.362 ^c^	0.369 ^c^	0.433 ^c^	0.598 ^c^	-									
M ^a^	-0.218 ^c^	0.191 ^c^	-0.311 ^c^	-0.240^c^	-0.174 ^c^	-0.155 ^c^	0.352 ^c^	0.362 ^c^	0.361 ^c^	0.481 ^c^	0.571 ^c^	0.596^c^	-								
N ^a^	-0.176 ^c^	0.215 ^c^	-0.202 ^c^	-0.160 ^c^	-0.143 ^c^	-0.201 ^c^	0.339 ^c^	0.469 ^c^	0.490^c^	0.480 ^c^	0.500^c^	0.548 ^c^	0.490^c^	-							
O ^a^	-0.003	0.163 ^c^	-0.007	-0.031	-0.002	-0.043 ^c^	0.179 ^c^	0.371 ^c^	0.300 ^c^	0.235 ^c^	0.272 ^c^	0.367 ^c^	0.402 ^c^	0.360 ^c^	-						
P ^a^	-0.345 ^c^	0.283 ^c^	-0.310 ^c^	-0.322 ^c^	-0.292 ^c^	-0.233 ^c^	0.481 ^c^	0.432 ^c^	0.463 ^c^	0.525 ^c^	0.588 ^c^	0.549^c^	0.638 ^c^	0.548 ^c^	0.432 ^b^	-					
Q ^a^	-0.047	0.069	-0.021	-0.007	-0.069	-0.066	0.097	0.187 ^c^	0.208 ^c^	0.096	0.064	0.161 c	0.009	0.244 ^c^	0.161 ^c^	0.186 ^c^	-				
R ^a^	-0.255 ^c^	0.199 ^c^	-0.159 ^c^	-0.255 ^c^	-0.300^c^	-0.126 ^b^	0.358 ^c^	0.137 ^b^	0.321 ^c^	0.417 ^c^	0.356 ^c^	0.316 ^c^	0.314 ^c^	0.291 ^c^	0.271 ^c^	0.396 ^c^	0.087	-			
S ^a^	-0.119 ^b^	0.133 ^b^	-0.023	-0.040	-0.108 ^b^	-0.080	0.168 ^c^	0.251 ^c^	0.264 ^c^	0.185 ^c^	0.147 ^c^	0.113 ^b^	0.014	0.126 ^b^	0.158 ^c^	0.142 ^c^	0.339 ^c^	0.259 ^c^	-		
T ^a^	-0.230 ^c^	0.177 ^b^	-0.152 ^c^	-0.207 ^c^	-0.239 ^c^	-0.157 ^c^	0.322 ^c^	0.315 ^c^	0.411 ^c^	0.356 ^c^	0.286 ^c^	0.199 ^c^	0.263 ^c^	0.342 ^c^	0.244 ^b^	0.337 ^c^	0.241 ^c^	0.348 ^c^	0.468 ^c^		
U ^a^	-0.256 ^c^	0.199 ^c^	-0.261 ^c^	-0.260^c^	-0.272 ^c^	-0.263 ^c^	0.350 ^c^	0.359 ^c^	0.364 ^c^	0.364 ^c^	0.398 ^c^	0.483 ^c^	0.414 ^c^	0.421 ^c^	0.308 ^c^	0.498 ^c^	0.245 ^c^	0.378 ^c^	0.205 ^c^	0.487 ^c^	-

^a^A, care; B, over protection; C, positive affect; D, father involvement; E, communicate; F, anger; G, emotional deprivation; H, abandonment/instability; I, mistrust/abuse; J, social isolation/alienation; K, defectiveness/shame; L, failure; M, dependence/incompetence; N, vulnerability to harm or illness; O, enmeshment/undeveloped self; Q, self-sacrifice; R, emotional inhibition; S, unrelenting standards; T, entitlement/grandiosity; U, insufficient self-control

^b^*P* < 0.05

^c^*P* < 0.01

**Table 2.** Stepwise Regression Analysis of Predictive Variables of Internalizing Behaviors

	F ^a^	Adjusted R Square	B	S.E ^a^	β	*t*-Test	*P* value
**Emotional deprivation**	45.879	0.207					
Care			-0.190	0.061	-0.230	-3.088	0.002
Father involvement			-0.235	0.067	-0.260	-3.490	0.001
**Abandonment/** **instability**	4.030	0.009					
Overprotection			0.119	0.059	0.108	2.007	0.045
**Enmeshment/** **undeveloped self**	9.375	0.024					
Overprotection			0.200	0.065	0.163	3.062	0.002
**Unrelenting standards/** **hypercriticalness**	6.152	0.015					
Overprotection			0.181	0.073	0.133	2.480	0.014
**Mistrust/abuse**	18.640	0.093					
Overprotection			0.181	0.047	0.214	3.858	0.000
Father involvement			-0.130	0.044	-0.162	-2.931	0.004
**Social Isolation/alienation**	23.887	0.117					
Overprotection			0.123	0.041	0.162	2.964	0.003
Father involvement			-0.182	0.039	-0.255	-4.667	0.000
**Defectiveness/shame**	27.675	0.134					
Overprotection			0.114	0.036	0.171	3.154	0.002
Father involvement			-0.173	0.034	-0.274	-5.053	0.000
**Failure**	32.796	0.085					
Positive affect			-0.142	0.025	-0.295	-5.727	0.000
**Dependence/incompetence**	36.817	0.094					
Positive affect			-0.128	0.021	-0.311	-6.068	0.000
**Vulnerability to harm or illness**	11.743	0.059					
Positive affect			-0.067	0.026	-0.144	-2.569	0.011
Overprotection			0.128	0.044	0.163	2.915	0.004
**Subjugation**	28.094	0.136					
Care			-0.187	0.038	-0.274	-4.938	0.000
Overprotection			0.131	0.044	0.165	2.978	0.003
**Emotional inhibition**	33.802	0.087					
Communicate			-0.254	0.044	-0.300	-5.814	0.000
**Entitlement/grandiosity**	20.857	0.055					
Communicate			-0.192	0.042	-0.239	-4.567	0.000
**Insufficient self-control/self-discipline**	17.825	0.089					
Communicate			-0.137	0.045	-0.185	-3.077	0.002
Anger			-0.581	0.209	-0.167	-2.78	0.006

^a^ Abbreviations: F, frequency; S.E, standard error

## 5. Discussion

The present research findings showed that father care, father involvement, positive affect, and an effective relationship with the father negatively correlated with all EMSs, whereas father excessive support positively correlated with all EMSs. In addition, these results showed that father-related variables had effective roles in predicting the variance of different schemas. The most remarkable point was that every aspect of the father’s relationship with their child played a special role indifferent EMS predictions. These findings were consistent with the concerns of many researchers about the roles of fathers in the psychopathology of children. The last two decades have witnessed a growing concern and interest in the role that fathers play in the life of their children (13-15), and, recently, many good studies have been conducted to model the links between fathers’ and children’s behaviors (16). Fathers can affect their children’s psychopathology in many ways, such as by their own psychopathology (17-19) and by their parenting styles (20, 21). Based on these research results, it is suggested that psychotherapists pay attention to restructuring the schemas for different aspects of father–child relationships with their patients. In addition, it is suggested that parental education be taught to fathers with educational packages according to the formula of psychologists and sociologists. The researchers hope that future studies examine how tomediatee and moderatee the variables in fathers that affect the psychopathology of children.
